# Supplementary material for: Hot spot prediction in protein-protein interactions by an ensemble system
Source: BMC Syst Biol. 2018 Dec 31;12(Suppl 9):132. doi: 10.1186/s12918-018-0665-8 (PMC6311905; doi:10.1186/s12918-018-0665-8)
Supplement: Supplementary file 2 — All relevant MATLAB code. A simple Matlab implement of our predictor. (ZIP 19,742 kb) [file 12918_2018_665_MOESM2_ESM.zip › Additional file 2/Read ASA/ASA Property/SKEMPI/1JRHH.txt]

 NetSurfP - Protein Surface Accessibility and Secondary Structure Predictions

  

|  |  |  |
| --- | --- | --- |
|  |  | NetSurfP - Protein Surface Accessibility and Secondary Structure Predictions  Technical University of Denmark |

  


---

```
# For publication of results, please cite:
# A generic method for assignment of reliability scores applied to solvent accessibility predictions.
# Bent Petersen, Thomas Nordahl Petersen, Pernille Andersen, Morten Nielsen and Claus Lundegaard
# BMC Structural Biology 2009, 9:51 doi:10.1186/1472-6807-9-51
#
# Column 1: Class assignment - B for buried or E for Exposed - Threshold: 25% exposure, but not based on RSA
# Column 2: Amino acid
# Column 3: Sequence name
# Column 4: Amino acid number
# Column 5: Relative Surface Accessibility - RSA
# Column 6: Absolute Surface Accessibility
# Column 7: Z-fit score for RSA prediction
# Column 8: Probability for Alpha-Helix
# Column 9: Probability for Beta-strand
# Column 10: Probability for Coil
E A  1JRHH                  1    0.724  79.763   0.325   0.003   0.003   0.994
B V  1JRHH                  2    0.194  29.833  -0.440   0.004   0.616   0.381
E K  1JRHH                  3    0.423  86.991   1.135   0.002   0.816   0.182
B L  1JRHH                  4    0.138  25.323   0.889   0.002   0.816   0.182
E Q  1JRHH                  5    0.391  69.868   0.638   0.002   0.816   0.182
B E  1JRHH                  6    0.246  43.064   0.400   0.004   0.514   0.481
E S  1JRHH                  7    0.410  48.064  -0.375   0.004   0.197   0.799
E G  1JRHH                  8    0.492  38.697  -1.778   0.018   0.047   0.935
E P  1JRHH                  9    0.532  75.548  -2.398   0.018   0.047   0.935
E G  1JRHH                 10    0.506  39.791  -1.960   0.019   0.141   0.840
E I  1JRHH                 11    0.378  70.004  -0.789   0.022   0.359   0.619
E L  1JRHH                 12    0.282  51.689   0.573   0.021   0.451   0.528
E K  1JRHH                 13    0.384  78.968   1.071   0.020   0.205   0.775
E P  1JRHH                 14    0.451  63.983  -0.660   0.005   0.045   0.951
E S  1JRHH                 15    0.601  70.449  -1.966   0.005   0.045   0.951
E Q  1JRHH                 16    0.456  81.424  -0.530   0.004   0.138   0.858
E T  1JRHH                 17    0.430  59.627  -0.411   0.004   0.420   0.576
B L  1JRHH                 18    0.127  23.327   0.803   0.002   0.816   0.182
E S  1JRHH                 19    0.424  49.740   0.971   0.001   0.900   0.099
B L  1JRHH                 20    0.072  13.275   0.620   0.001   0.900   0.099
B T  1JRHH                 21    0.182  25.243   0.842   0.001   0.900   0.099
B C  1JRHH                 22    0.047   6.557   0.409   0.001   0.900   0.099
B S  1JRHH                 23    0.293  34.375   0.464   0.001   0.900   0.099
B F  1JRHH                 24    0.091  18.304  -0.343   0.002   0.816   0.182
B S  1JRHH                 25    0.188  22.069  -0.629   0.004   0.420   0.576
E G  1JRHH                 26    0.260  20.478  -1.607   0.004   0.138   0.858
B F  1JRHH                 27    0.219  43.893  -0.741   0.021   0.279   0.699
B S  1JRHH                 28    0.121  14.216  -0.737   0.022   0.552   0.426
B L  1JRHH                 29    0.077  14.044  -0.652   0.022   0.552   0.426
B T  1JRHH                 30    0.136  18.808  -1.636   0.022   0.359   0.619
B T  1JRHH                 31    0.188  26.076  -1.951   0.019   0.141   0.840
B Y  1JRHH                 32    0.184  39.235  -1.545   0.018   0.047   0.935
B G  1JRHH                 33    0.204  16.047  -1.242   0.018   0.088   0.893
B M  1JRHH                 34    0.109  21.751  -0.159   0.021   0.279   0.699
B G  1JRHH                 35    0.118   9.287  -0.516   0.021   0.451   0.528
B V  1JRHH                 36    0.069  10.590  -0.188   0.022   0.552   0.426
B G  1JRHH                 37    0.051   3.998  -0.558   0.022   0.552   0.426
B W  1JRHH                 38    0.033   7.840   0.288   0.023   0.655   0.322
B I  1JRHH                 39    0.028   5.180   0.470   0.021   0.756   0.223
B R  1JRHH                 40    0.096  22.007   0.367   0.023   0.655   0.322
B Q  1JRHH                 41    0.075  13.466   0.239   0.022   0.359   0.619
B S  1JRHH                 42    0.211  24.682   0.668   0.005   0.045   0.951
E S  1JRHH                 43    0.337  39.520   0.556   0.005   0.015   0.979
E G  1JRHH                 44    0.516  40.641  -1.468   0.018   0.019   0.964
E K  1JRHH                 45    0.371  76.335   0.533   0.018   0.019   0.964
E G  1JRHH                 46    0.403  31.716  -0.411   0.018   0.088   0.893
B L  1JRHH                 47    0.144  26.348   1.001   0.005   0.336   0.660
B E  1JRHH                 48    0.105  18.309  -0.041   0.003   0.718   0.279
B W  1JRHH                 49    0.045  10.895   0.066   0.001   0.900   0.099
B L  1JRHH                 50    0.025   4.632   0.856   0.001   0.959   0.040
B A  1JRHH                 51    0.018   2.017  -0.194   0.001   0.959   0.040
B H  1JRHH                 52    0.095  17.335   0.203   0.001   0.959   0.040
B I  1JRHH                 53    0.031   5.698   0.116   0.001   0.959   0.040
B W  1JRHH                 54    0.187  44.974   0.646   0.003   0.718   0.279
E W  1JRHH                 55    0.377  90.789  -1.039   0.004   0.197   0.799
E D  1JRHH                 56    0.587  84.587  -2.158   0.005   0.015   0.979
E D  1JRHH                 57    0.449  64.672  -2.031   0.018   0.019   0.964
E D  1JRHH                 58    0.507  72.987  -0.675   0.018   0.047   0.935
E K  1JRHH                 59    0.367  75.533  -0.266   0.019   0.141   0.840
E Y  1JRHH                 60    0.432  92.233   0.199   0.064   0.216   0.721
B Y  1JRHH                 61    0.218  46.565   0.321   0.056   0.142   0.802
B N  1JRHH                 62    0.250  36.527  -0.125   0.052   0.084   0.864
E P  1JRHH                 63    0.632  89.667  -0.599   0.053   0.043   0.903
E S  1JRHH                 64    0.389  45.579   0.134   0.053   0.043   0.903
B L  1JRHH                 65    0.095  17.358  -0.472   0.019   0.141   0.840
E K  1JRHH                 66    0.382  78.660   0.707   0.020   0.205   0.775
B S  1JRHH                 67    0.231  27.108  -1.515   0.005   0.336   0.660
B R  1JRHH                 68    0.217  49.739   0.811   0.003   0.718   0.279
B L  1JRHH                 69    0.057  10.400  -0.310   0.001   0.900   0.099
B T  1JRHH                 70    0.128  17.726   0.614   0.001   0.959   0.040
B I  1JRHH                 71    0.047   8.769   0.577   0.001   0.959   0.040
B S  1JRHH                 72    0.123  14.462   0.173   0.002   0.816   0.182
E K  1JRHH                 73    0.271  55.827   0.898   0.004   0.616   0.381
B D  1JRHH                 74    0.246  35.449  -0.505   0.004   0.420   0.576
E T  1JRHH                 75    0.442  61.278  -0.073   0.018   0.088   0.893
E S  1JRHH                 76    0.278  32.593  -1.549   0.018   0.088   0.893
E R  1JRHH                 77    0.544 124.507   0.967   0.019   0.141   0.840
E N  1JRHH                 78    0.268  39.279  -0.417   0.005   0.336   0.660
B Q  1JRHH                 79    0.244  43.507   1.225   0.002   0.816   0.182
B V  1JRHH                 80    0.077  11.789  -0.549   0.006   0.962   0.032
B F  1JRHH                 81    0.190  38.213   0.364   0.001   0.959   0.040
B L  1JRHH                 82    0.050   9.173   0.732   0.001   0.959   0.040
B K  1JRHH                 83    0.228  46.838   0.127   0.001   0.900   0.099
B I  1JRHH                 84    0.096  17.797  -0.296   0.004   0.616   0.381
E T  1JRHH                 85    0.411  57.033  -0.158   0.004   0.420   0.576
E S  1JRHH                 86    0.407  47.759  -0.671   0.004   0.197   0.799
B V  1JRHH                 87    0.123  18.874   0.252   0.004   0.085   0.910
E A  1JRHH                 88    0.481  52.973   1.215   0.018   0.047   0.935
E T  1JRHH                 89    0.386  53.580  -0.693   0.052   0.084   0.864
E A  1JRHH                 90    0.435  47.959  -0.660   0.053   0.043   0.903
B D  1JRHH                 91    0.185  26.630  -0.884   0.052   0.084   0.864
B T  1JRHH                 92    0.125  17.310   0.934   0.069   0.386   0.545
B A  1JRHH                 93    0.042   4.639  -0.085   0.021   0.756   0.223
B T  1JRHH                 94    0.070   9.709   0.987   0.018   0.846   0.136
B Y  1JRHH                 95    0.036   7.650   0.953   0.018   0.846   0.136
B Y  1JRHH                 96    0.052  11.070   0.839   0.018   0.846   0.136
B C  1JRHH                 97    0.016   2.246   1.554   0.021   0.756   0.223
B A  1JRHH                 98    0.027   2.942   0.281   0.023   0.655   0.322
B R  1JRHH                 99    0.153  34.968   0.276   0.021   0.451   0.528
B R  1JRHH                100    0.255  58.464  -0.326   0.021   0.279   0.699
B A  1JRHH                101    0.299  32.972  -0.595   0.018   0.088   0.893
E P  1JRHH                102    0.428  60.776  -2.045   0.018   0.088   0.893
E F  1JRHH                103    0.390  78.273  -2.130   0.018   0.088   0.893
E Y  1JRHH                104    0.319  68.170  -1.851   0.018   0.088   0.893
E G  1JRHH                105    0.371  29.190  -1.858   0.018   0.047   0.935
E N  1JRHH                106    0.357  52.323  -1.878   0.018   0.047   0.935
E H  1JRHH                107    0.305  55.570  -1.029   0.018   0.047   0.935
B A  1JRHH                108    0.117  12.871  -0.763   0.019   0.141   0.840
B M  1JRHH                109    0.060  11.926  -0.213   0.020   0.205   0.775
B D  1JRHH                110    0.095  13.661  -0.680   0.020   0.205   0.775
B Y  1JRHH                111    0.078  16.626  -0.622   0.021   0.279   0.699
B W  1JRHH                112    0.048  11.592  -0.576   0.020   0.205   0.775
B G  1JRHH                113    0.052   4.053  -1.549   0.019   0.141   0.840
B Q  1JRHH                114    0.214  38.167   0.254   0.004   0.085   0.910
B G  1JRHH                115    0.143  11.270  -1.427   0.004   0.197   0.799
B T  1JRHH                116    0.084  11.692  -0.031   0.004   0.420   0.576
E T  1JRHH                117    0.282  39.155   0.047   0.002   0.816   0.182
B V  1JRHH                118    0.054   8.300   0.610   0.001   0.900   0.099
B T  1JRHH                119    0.121  16.727   0.110   0.011   0.918   0.071
B V  1JRHH                120    0.092  14.187   0.674   0.018   0.846   0.136
B S  1JRHH                121    0.204  23.921  -0.377   0.004   0.514   0.481
B S  1JRHH                122    0.271  31.749  -0.991   0.020   0.205   0.775
E A  1JRHH                123    0.438  48.301  -1.706   0.018   0.047   0.935
E K  1JRHH                124    0.446  91.783   0.020   0.018   0.047   0.935
E T  1JRHH                125    0.482  66.923  -0.603   0.018   0.088   0.893
E T  1JRHH                126    0.590  81.805  -0.698   0.018   0.047   0.935
E P  1JRHH                127    0.421  59.797  -0.850   0.005   0.015   0.979
B C  1JRHH                128    0.216  30.256  -0.651   0.005   0.045   0.951
E L  1JRHH                129    0.570 104.294  -0.583   0.004   0.138   0.858
B V  1JRHH                130    0.163  24.992  -1.065   0.005   0.262   0.733
B Y  1JRHH                131    0.196  41.864   0.643   0.004   0.138   0.858
B F  1JRHH                132    0.280  56.256  -0.424   0.005   0.045   0.951
B P  1JRHH                133    0.221  31.317  -1.301   0.018   0.047   0.935
E E  1JRHH                134    0.494  86.389  -1.155   0.018   0.047   0.935
E P  1JRHH                135    0.408  57.966  -1.854   0.004   0.085   0.910
B V  1JRHH                136    0.109  16.723  -0.980   0.005   0.262   0.733
E T  1JRHH                137    0.448  62.165  -0.843   0.004   0.514   0.481
B V  1JRHH                138    0.270  41.438  -0.321   0.022   0.552   0.426
E T  1JRHH                139    0.452  62.679  -0.749   0.021   0.451   0.528
B W  1JRHH                140    0.213  51.323  -0.235   0.022   0.359   0.619
E N  1JRHH                141    0.562  82.262  -0.843   0.004   0.197   0.799
E S  1JRHH                142    0.533  62.479  -2.349   0.018   0.047   0.935
E G  1JRHH                143    0.680  53.516  -2.353   0.018   0.019   0.964
E S  1JRHH                144    0.449  52.564  -2.169   0.018   0.047   0.935
E S  1JRHH                145    0.524  61.448  -0.977   0.018   0.088   0.893
E G  1JRHH                146    0.384  30.229  -0.925   0.005   0.336   0.660
B V  1JRHH                147    0.224  34.506  -0.328   0.003   0.718   0.279
E H  1JRHH                148    0.317  57.608   0.268   0.002   0.816   0.182
E T  1JRHH                149    0.359  49.835  -0.577   0.021   0.756   0.223
B F  1JRHH                150    0.203  40.702   0.848   0.021   0.451   0.528
B P  1JRHH                151    0.299  42.371   0.076   0.021   0.279   0.699
B A  1JRHH                152    0.265  29.225  -1.411   0.022   0.359   0.619
B V  1JRHH                153    0.247  37.902  -0.138   0.021   0.451   0.528
E L  1JRHH                154    0.351  64.286  -0.429   0.022   0.359   0.619
E Q  1JRHH                155    0.554  98.909  -0.728   0.020   0.205   0.775
E S  1JRHH                156    0.711  83.353  -1.760   0.018   0.047   0.935
E D  1JRHH                157    0.550  79.212  -2.398   0.005   0.045   0.951
E L  1JRHH                158    0.392  71.812  -0.271   0.004   0.420   0.576
B Y  1JRHH                159    0.182  38.829  -0.523   0.003   0.718   0.279
B T  1JRHH                160    0.219  30.389  -1.256   0.002   0.816   0.182
B L  1JRHH                161    0.178  32.519  -1.046   0.002   0.816   0.182
B S  1JRHH                162    0.190  22.303  -0.556   0.002   0.816   0.182
B S  1JRHH                163    0.155  18.178  -0.847   0.002   0.816   0.182
E S  1JRHH                164    0.321  37.656  -0.379   0.001   0.900   0.099
B V  1JRHH                165    0.079  12.204   0.443   0.001   0.959   0.040
E T  1JRHH                166    0.393  54.495   1.296   0.001   0.959   0.040
B C  1JRHH                167    0.040   5.574   0.089   0.001   0.959   0.040
E N  1JRHH                168    0.465  68.091   1.304   0.001   0.900   0.099
B V  1JRHH                169    0.071  10.928  -0.198   0.011   0.918   0.071
E A  1JRHH                170    0.593  65.316   0.732   0.002   0.816   0.182
B H  1JRHH                171    0.178  32.324   0.420   0.004   0.514   0.481
E P  1JRHH                172    0.627  88.957   0.239   0.018   0.047   0.935
E A  1JRHH                173    0.646  71.200  -1.051   0.018   0.019   0.964
E S  1JRHH                174    0.561  65.749  -0.798   0.018   0.047   0.935
E S  1JRHH                175    0.646  75.676  -0.067   0.018   0.088   0.893
E T  1JRHH                176    0.498  69.045   0.823   0.020   0.205   0.775
E K  1JRHH                177    0.571 117.475   1.164   0.004   0.420   0.576
E V  1JRHH                178    0.332  50.982  -0.204   0.004   0.420   0.576
E D  1JRHH                179    0.637  91.777  -0.163   0.021   0.279   0.699
E K  1JRHH                180    0.800 164.498   1.065   0.003   0.003   0.994

Explain the output.  Go back.
```

---
